# MSF-HierGNN: a multi-source substructure-fusion hierarchical GNN method and web server to predict molecular property for drug design

**DOI:** 10.1093/bib/bbag491

**Published:** 2026-09-10

**Authors:** Ming Xiao, Yi Xiao, Yingping Wu, Yujie You, Xiaolei Liu, Hoang Van Thanh, Le Zhang

**Affiliations:** College of Computer Science, Sichuan University, No. 24 South Section 1, Yihuan Road, Wuhou District, Chengdu 610065, China; College of Computer Science, Sichuan University, No. 24 South Section 1, Yihuan Road, Wuhou District, Chengdu 610065, China; College of Computer Science, Sichuan University, No. 24 South Section 1, Yihuan Road, Wuhou District, Chengdu 610065, China; School of Computer Science and Engineering, Sichuan University of Science and Engineering, No. 1 Baita Road, Sanjiang New Area, Yibin 644000, China; Department of Geriatrics and National Clinical Research Center for Geriatrics, West China Hospital, Sichuan University, No. 37 Guoxue Alley, Wuhou District, Chengdu, Sichuan 610041, China; College of Mechanical Engineering, Vietnam Maritime University, No. 484 Lach Tray Street, Le Chan District, Hai Phong 180000, Vietnam; College of Computer Science, Sichuan University, No. 24 South Section 1, Yihuan Road, Wuhou District, Chengdu 610065, China

**Keywords:** molecular property prediction, graph neural network, substructure representation, multi-source information fusion, drug discovery and target prediction

## Abstract

Accurate prediction of the biological and physicochemical properties of molecules is of great significance in shortening the process and decreasing the failure rate of drug design. Thus, previous studies have established several benchmark datasets and developed several Graph Neural Networks (GNNs) based artificial intelligence (AI) predictive methods. However, since these methods encounter challenges such as incomplete representation of molecular hierarchical structures, insufficient exploration of local topological features, and under-extraction of correlations among atomic features, our study proposes a graph neural network that combines hierarchical pooling with localized substructure modeling named MSF-HierGNN (Multi-Source Substructure-Fusion Hierarchical Graph Neural Network) to solve these problems. Firstly, MSF-HierGNN constructs a more comprehensive and multiscale graph-level representation by preserving chemically salient structures and fusing multiple sources of substructure. Secondly, the model can more comprehensively represent molecular substructure features by integrating multiple fragmentation algorithms and incorporating five molecular fingerprints. Additionally, the model further captures correlations among atomic features by increasing the message-passing mechanism. Finally, we validate the model's effectiveness using public benchmark datasets and develop an interactive and user-friendly web server application based on MSF-HierGNN. Experiments based on benchmark datasets demonstrate that our model not only can effectively extract molecular substructure features and capture correlations among atomic features, but also can more accurately predict molecular properties, thereby offering a novel AI method and application to support drug discovery and design.

## Introduction

The clinical efficacy and safety of a drug are fundamentally determined by its molecular properties and target characteristics [1], but it is costly and extremely time-consuming [2] for us to evaluate the physicochemical, pharmacokinetic, and toxicological properties of candidate compounds by classical experimental methods. Because computational predictive methods can not only enable the rapid evaluation of drug molecule properties at very low cost, but also guide the targeted optimization of lead compounds during drug design [3], the development of accurate artificial intelligence (AI)-based molecular properties predictive methods and applications is of great significance in shortening the process and decreasing the failure rate of drug design.

Since molecular properties encompass multiple key features such as water solubility [4], biological activity [5], molecular toxicity [6], and free energy [7], previous studies have established several representative benchmark datasets to assist in predicting these molecular properties. In recent years, Graph Neural Networks (GNNs) have demonstrated great potential in molecular property prediction [8]. A key advantage of GNNs lies in their natural ability to represent molecules as graphs, where atoms correspond to nodes and chemical bonds correspond to edges [9–11]. This representation not only facilitates capturing topological structures and interatomic interactions more effectively, but also enables iterative aggregation of neighboring node information to update node representations, thereby offering a comprehensive description for molecular structures and increasing predictive performance [9]. Nevertheless, despite the progress of GNNs in molecular property prediction, there are still areas for improvement in graph-level representation, substructure extraction, and message passing.

Firstly, in terms of graph-level representation, studies such as Yang *et al.* [12] employed global pooling strategy and generated a graph representation by aggregating the features of all atoms to enable graph-level prediction. However, such strategies may dilute or lose critical feature information from key atoms during the pooling process. To decrease the information loss caused by global pooling, studies like Kong *et al.* [13] employed hierarchical pooling strategy that progressively coarsens the graph structure, thereby preserving the hierarchical information of molecules. However, these studies usually used a predefined graph coarsening algorithm and aggregated only a limited set of key substructure types, making it difficult to represent a more comprehensive molecular hierarchy. Therefore, how to emphasize the key structural features and make a more complete hierarchical graph-level representation of molecules to increase the accuracy of GNN-based drug molecular property prediction becomes our first scientific question.

Second, in terms of substructure extraction, many previous studies, like Cai *et al.* [14] used molecular fingerprints to directly capture several chemical characteristics and represented these as substructures with specific chemical semantics to serve as auxiliary inputs to GNN models, thereby increasing the predictive accuracy. However, molecular fingerprints are often one-dimensional binary sequences and difficult to fully describe molecular topology, which imposes certain limitations [15]. To complement the local topological features of molecules, Zhu *et al.* [16] employed the Breaking of Retrosynthetically Interesting Chemical Substructures (BRICS) algorithm [17] algorithm to extract chemical subgraphs that represent molecular topological substructures, and then fused the feature representations of these subgraphs with whole-graph feature representations to achieve better predictive performance. However, rule-based subgraph partitioning algorithms often generate large molecular fragments that are only useful for a limited set of tasks [18]. Therefore, how to complementarily fuse the chemical-semantic features carried by molecular fingerprints with the local topological features derived from subgraph partitioning, to further increase the predictive accuracy becomes our second scientific question.

Third, in terms of GNN message passing, Duvenaud *et al.* [19] introduced the GCN graph convolution operators, which predict molecular properties by performing combined updates on the features of atoms and their neighbors within the molecular graph. However, GCN convolutions primarily update atomic features based on atoms, resulting in insufficient utilization of chemical bonds. To better explore the role of chemical bonds in molecular property prediction, Yang *et al*. [20] introduced bond features and proposed a directed edge message passing mechanism. However, since this method cannot distinguish the relative importance of different edges, it is not only hard to reflect the differences between various chemical bonds in molecular graphs, but also leads to certain inaccuracies [21]. Therefore, our third scientific question is how to further capture correlations among atomic features by incorporating the differential importance of chemical bonds during GNN message passing.

Finally, although previous GNN-based studies [14, 19–21] mostly visualized and compared results such as molecular graph substructure and atomic feature correlations, seldom interactive, and user-friendly platforms for visualizing and analyzing molecular property predictions across multiple typical benchmark datasets have been developed. Thus, we propose the following innovations to solve the questions outlined above.

Firstly, we develop a graph neural network that combines hierarchical pooling with localized substructure modeling named MSF-HierGNN (Multi-Source Substructure-Fusion Hierarchical Graph Neural Network) to emphasize the key structural features and make a more complete hierarchical graph-level representation of molecules. By progressively filtering important atoms layer by layer in the molecular graph, the model can extract more comprehensive hierarchical information from the graph, thereby resulting more accurate graph-level representation.

Secondly, to obtain a substructure representation that both keep chemical semantics and reflect molecular topology for predictions, we firstly partition molecules into local topological substructure by fusing multiple fragmentation algorithms, including BRICS [17], Murcko scaffold boundary detection [22], ring based cutting [23], and functional group based cutting [24]. And then, we introduce five molecular fingerprints to capture chemically meaningful substructures, thereby providing a more comprehensive representation of molecular substructure features.

Third, to further capture correlations among atomic features, the model performs weighted aggregation of these features to increase its ability to distinguish the importance of different chemical bonds and the predictive accuracy.

Finally, we validate the effectiveness of the proposed model by publicly available datasets for drug-related molecular property prediction, and developed an interactive and user-friendly web server application for visualizing and analyzing molecular property predictions based on the MSF-HierGNN algorithm.

The experiments demonstrate that MSF-HierGNN statistically significantly outperforms most of the eleven baseline models, which proves that MSF-HierGNN can not only effectively extract molecular substructure features to guide the design of molecules targeting specific proteins, but also predict molecular properties more accurately to avoid failures in drug design. Also, the ablation study shows that removing any module from MSF-HierGNN will statistically significantly decrease its predictive accuracy, which turns out the innovation and effectiveness of the molecular substructure representation module and the structure aware hierarchical pooling module. As shown by the visual comparative analysis provided through our web server application, our improved message-passing mechanism not only can more accurately reflect differences in molecular chemical bonds within graph neural networks, but also can capture the correlations among atomic features, thereby offering a novel AI method and application to support drug discovery and design.

## Materials and methods

### Dataset

To compare our method with previous studies, we carried out molecular property predictive experiments on eight commonly used benchmark datasets from MoleculeNet [5], including five classification datasets: beta-site amyloid precursor protein cleaving enzyme (BACE) [5], blood-brain barrier penetration (BBBP) [25], Tox21 [6], Side Effect Resource (SIDER) [26], and ClinTox [5], and three regression datasets: estimated solubility (ESOL) [4], FreeSolv [7], and Lipophilicity [27]. All datasets were randomly split into training, validation, and test sets in an 8: 1:1 ratio, but with a distinction in splitting strategy: for BACE and BBBP, we used scaffold splitting to prevent structural overlapping between training and test molecules, thereby providing a more stringent evaluation of generalization ability. For the remaining six datasets, random splitting was applied because their molecular structures are more diverse and random partition is the standard practice in prior studies for these tasks.

These datasets span multiple research areas such as physical chemistry, toxicology, and pharmacokinetics [28]. Detailed information is listed in Supplementary Table S1.

### Multi-source substructure-fusion hierarchical graph neural network (MSF-HierGNN)

**Figure 1:**
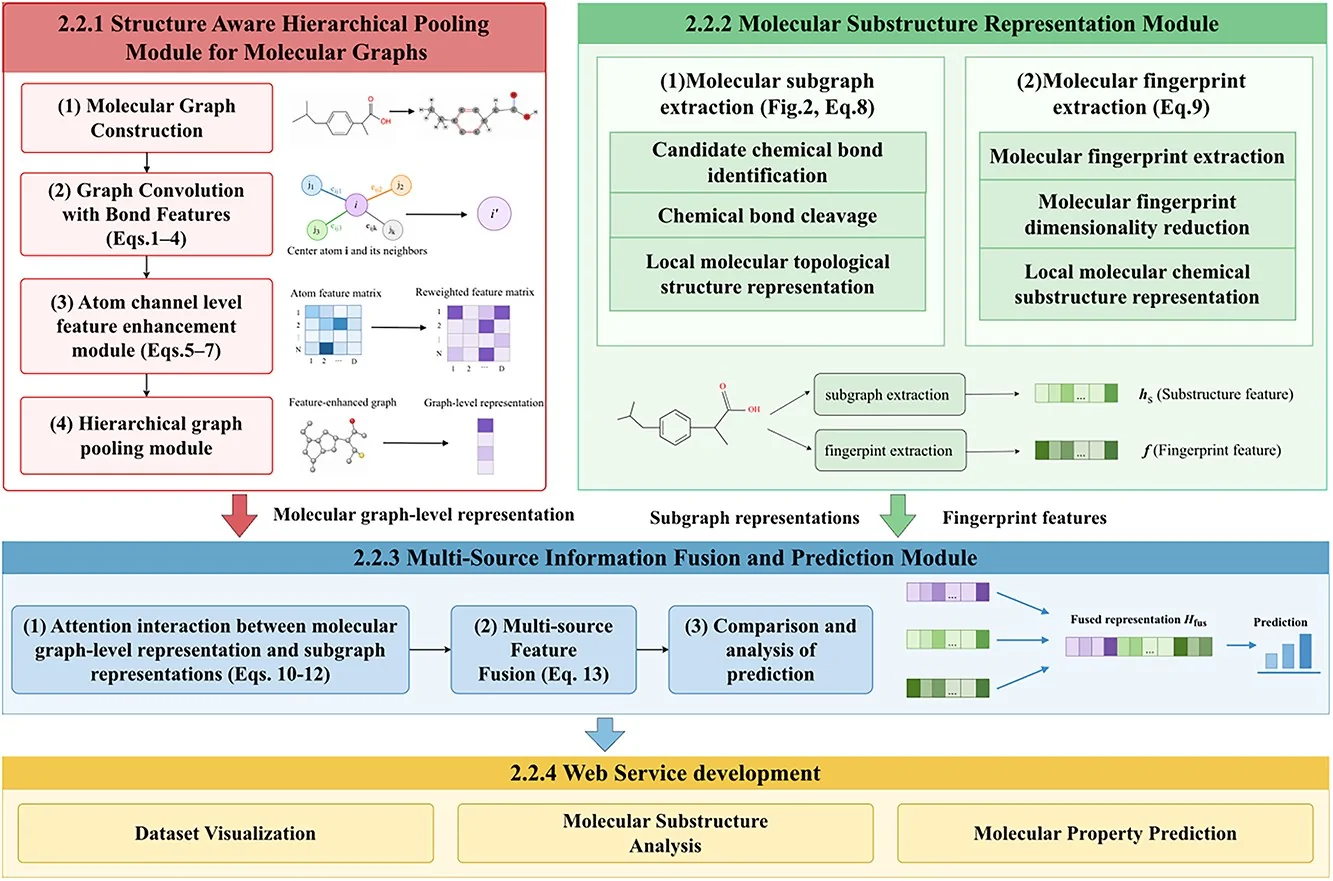
Multi-source substructure-fusion hierarchical graph neural network (MSF-HierGNN).

As shown in Fig. 1, MSF-HierGNN model consists of three principal modules: structure aware hierarchical pooling module for molecular graphs, molecular substructure representation module, and multi-source information fusion and prediction module.

First, we process the global molecular graph using a structure aware hierarchical pooling module to highlight key atoms within the graph and obtain the hierarchical molecular graph-level representations (Supplementary Fig. S1). Second, the molecular substructure representation module extracts both subgraph topologies and molecular fingerprints, thereby characterizing the molecule’s local topological patterns and substructures with explicit chemical semantics (Supplementary Fig. S2). Third, to reflect the relationships between subgraph and global topology, we develop a cross-scale attention interactive mechanism which can obtain more effective feature embeddings by interacting subgraph level with graph level feature representations. Finally, the graph level representation, subgraph representations, and fingerprint features are fused, and then they are fed into the predictive module to generate molecular property predictions. The key steps are detailed below.

#### Structure aware hierarchical pooling module for molecular graphs

##### Molecular graph construction

First, we converted the SMILES sequences of the dataset into molecular graphs by RDKit [29], and then initialized atomic and chemical bond features [19]. The properties covered by atomic and chemical bond features are listed by Supplementary Table S2.

##### Graph convolution with bond features

Second, to address the question that existing graph convolution do not consider the difference of chemical bonds, we extend a commonly used graph convolution operator by introducing inter-atomic difference and similarity terms [30], which allows weighted aggregation of bond features and thus enables better learning of atom and bond representations for molecular graphs.

Concretely, we firstly construct an atom-interaction vector ${\mathrm{h}}_{\mathrm{ij}}$ (Eq. 1) for a given atom.


(1) \begin{equation*} {h}_{ij}=\left[{W}_i{x}_i+{W}_j{x}_j\parallel \left({x}_j-{x}_i\right)\parallel \left({x}_j\odot{x}_i\right)\right]\kern7.25em \end{equation*}

Here, ${x}_i$ and ${x}_j$ represent the feature vectors of atoms ${v}_i$and${v}_j$, respectively; ${W}_i$ and ${W}_j$ are learnable weight matrices; *∥* represents the feature concatenation and *⊙* indicates element-by-element multiplication. The atom-interaction vector ${\mathrm{h}}_{\mathrm{ij}}$ encodes the association strength between atoms, which often affects chemical bond strength [31].

Accordingly, we use the atom-interaction vector to derive the relative importance of different chemical bonds. We simultaneously capture an atom’s intrinsic attributes, inter-atomic heterogeneity, and inter-atomic homophilic cooperation by concatenating the atom features, the inter-atomic difference term, and the similarity term, thereby representing the pairwise association between atoms. Subsequently, to model the amplification or suppression of information exchanged between atom pairs, we compute an attention score ${\alpha}_{ij}$ for each atom pair based on the atom-interaction vector (Eq. 2).


(2) \begin{equation*} {\alpha}_{ij}=\mathit{\tanh}\left({h}_{ij}\right)\in \left(-1,1\right) \end{equation*}

Here, tanh is the hyperbolic tangent activation function. Finally, we construct the message ${m}_{ij}$ (Eq. 3) passing between atoms and update the atom representation ${x}_i^{\prime }$ (Eq. 4).


(3) \begin{equation*} {m}_{ij}={\alpha}_{ij}\cdotp{W}_{ij}\left(\left[{x}_j\parallel{e}_{ij}\parallel{x}_i\right]\right) \end{equation*}

(4) \begin{equation*} {x}_i^{\prime }=\left(1+\epsilon \right){x}_i+\sum_{j\in \mathcal{N}(i)}{m}_{ij} \end{equation*}

Here, ${\mathrm{e}}_{\mathrm{ij}}$ represents the edge feature between atoms ${\mathrm{v}}_{\mathrm{i}}$ and ${\mathrm{v}}_{\mathrm{j}}$, *ε* is a learnable scalar hyperparameter and ${\mathrm{W}}_{\mathrm{ij}}$ is a learnable weight matrix. $j\in \mathcal{N}(i)$ represents the index of a neighbor atom of ${v}_i$.

##### Atom channel level feature enhancement module

After graph convolution, in order to highlight the key atoms in the molecular graph, inspired by Squeeze-and-Excitation [32] we improved the atom channel level feature enhancement module to make it more suitable for graph structures. Specifically, we firstly capture both the overall average information and salient features of the molecular graph by computing the average and max pooling components for the graph-level semantic vector *s* (Eq. 5).


(5) \begin{equation*} s={w}_0\cdotp \frac{1}{N}\sum_{u=1}^N{x}_u+{w}_1\cdotp \underset{u=1,\dots, N}{\mathit{\max}}{x}_u \end{equation*}

Here, ${w}_0$ and ${w}_1$ are learnable weights used to balance the global semantics from different perspectives. *N* denotes the total number of atoms in a single molecule, and ${x}_u$ is the feature vector of the u^th^ atom.

Secondly, we generate the attention coefficient for each channel by inputting the graph level vector *s* into two affine and nonlinear mappings (Eq. 6).


(6) \begin{equation*} a=\sigma \left({W}_2\ ReLU\left({W}_1s\right)\right) \end{equation*}

Here, ${\mathrm{W}}_1\in{\mathrm{R}}^{\mathrm{C}/\mathrm{r}\times \mathrm{C}},{\mathrm{W}}_2\in{\mathrm{R}}^{\mathrm{C}\times \mathrm{C}/\mathrm{r}}$. ${W}_1$ and ${W}_2$ are learnable matrices, *ReLU* is a rectified linear activation function. r represents the reduction ratio and C represents the number of channels. *σ* denotes the element-wise Sigmoid function. Finally, we broadcast the graph-level channel weights back to the atom level and multiply them channel-wise with the original atom features to obtain the output (Eq. 7).


(7) \begin{equation*} \overset{\sim }{x}={x}_i\odot a \end{equation*}

Here ⊙ indicates element-by-element multiplication.

##### Hierarchical graph pooling module

After the atom channel level features were augmented, we employ the Top-k strategy [33] to select key atoms, thereby obtaining a coarsened graph that keeps only a subset of critical atoms from the molecular graph. With multiple rounds of molecular graph coarsening, we obtain hierarchical representations for the molecular graph [33]. Finally, we fuse each layer of coarsened graph representations to obtain the graph-level representation of the molecule.

#### Molecular substructure representation module

Next, we employ the molecular substructure representation module to extract the topological structure of molecular subgraphs and fingerprints, which describe the local topological structure of molecules and provide chemically meaningful information about molecular substructures.

##### Molecular subgraph extraction

Since current molecular extraction algorithms often produce large fragments [18], it is difficult to represent local molecular topologies at finer granularity. To alleviate the issue, we integrate multiple chemical substructure extraction algorithms to identify a larger set of chemically cleavable candidate bonds from the molecular graph. After that, we take the union of these bonds and cut the molecular graph accordingly to obtain a set of molecular subgraphs. The resulting molecular subgraph set will yield molecular subgraph features after passing through the local topology processing step.

**Figure 2:**
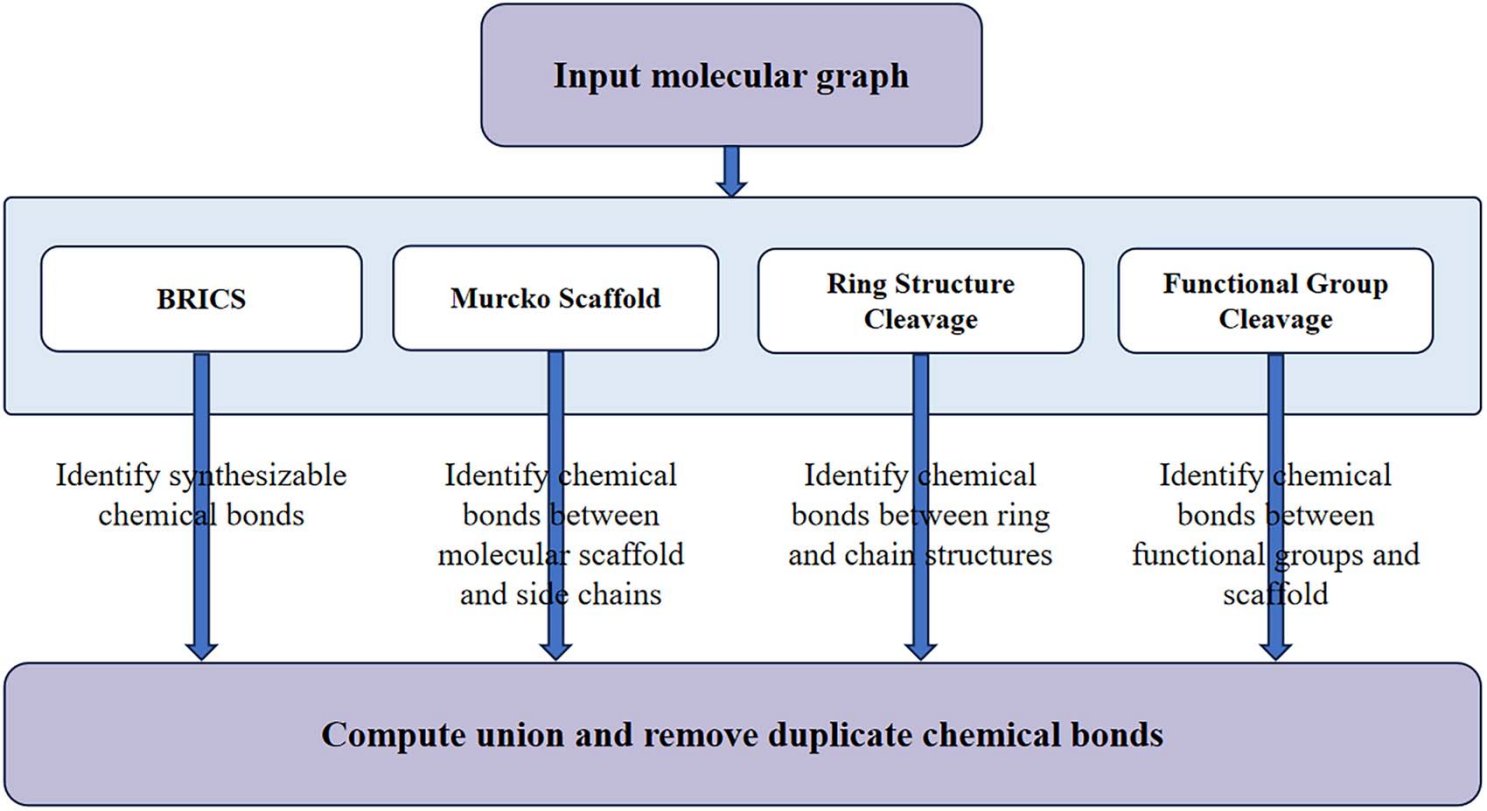
The process of identifying candidate cleavable bonds by combining multiple chemical substructure extraction algorithms.

Specifically, we firstly combine four chemical substructure extraction algorithms BRICS [17], Murcko Scaffold [22], ring-cleavage [34] and functional group based fragmentation [35], to identify candidate cleavable bonds and take the union of the results as Fig. 2. The rationale behind these fragmentation choices lies in their complementary chemical perspectives. BRICS generates fragments based on retro synthetically meaningful bond-breaking rules. Murcko scaffold extraction retains ring systems and linkers while removing side chains, which highlights the core molecular framework and helps us capture the global topological template of a molecule. Ring-based cutting further focuses on ring systems and their boundary structures, as cyclic motifs are often closely related to molecular rigidity, aromaticity, and drug-like properties. Functional group-based cutting, in contrast, targets local reactive moieties that are directly associated with physicochemical properties and biological activity. By integrating these different levels of fragmentation results, the model can simultaneously encode core scaffolds, ring structures, and synthetically meaningful fragments, thereby reducing the structural bias introduced by any single fragmentation strategy and improving the chemical interpretability of molecular substructure representations.

Secondly, we remove the corresponding edges in the graph according to the identified cleavable candidate bonds in the molecular graph of the bond cleavage step, and then cut them into a set of subgraphs denoted by ${G}^{\prime }$ (Eq. 8).


(8) \begin{equation*} {G}^{\prime }=\left\{{G}_1,{G}_1\dots{G}_n\right\} \end{equation*}

Here, ${G}_i$ is the i^th^ subgraph and n is the total number of subgraphs of the molecular graph.

Finally, we employ the message passing operator mentioned in *Structure Aware Hierarchical Pooling Module for Molecular Graphs* Section (Eqs. 3 and 4) to perform message passing within each subgraph, and then update the atom feature representations for the subgraph in the local topology representation step. After that, we apply a global pooling readout for each subgraph to have the molecular subgraph features.

##### Molecular fingerprint extraction

Molecular fingerprints can encode chemical functional substructures, which can be employed as auxiliary inputs for the GNN and supplement explicit chemical prior information. For example FP-GNN demonstrated that combining three complementary fingerprints in the FP-GNN architecture is statistically significant better than only using Morgan fingerprint [14]. For this purpose, we firstly select three complementary fingerprints that are consistent with FP-GNN: MACCS fingerprint [36], Morgan count fingerprint [37], and extended reduced graph (ERG) fingerprint [38]. On this basis, we added two classic molecular fingerprints: topological torsion fingerprint [39] and atomic pair fingerprint [40] to supplement more complementary information. By integrating these five representations, the model simultaneously accesses local substructures, local environments, backbone torsions, long-range topology, and pharmacophore information, thereby producing a more holistic molecular description than any single fingerprint alone. To solve the high dimensionality and sparsity problems after concatenating these fingerprints, we employ a linear projection to make dimensionality reduction and have the reduced fingerprint representation ${\textbf{f}}^{\prime }$ (Eq. 9).


(9) \begin{equation*} {\mathrm{f}}^{\prime }={\mathrm{W}}_{\mathrm{f}\mathrm{p}}\mathrm{f}+{\mathrm{b}}_{\mathrm{f}\mathrm{p}} \end{equation*}

Here, ${\mathrm{W}}_{\mathrm{fp}}$ represents a learnable matrix, ${\mathrm{b}}_{\mathrm{fp}}$ represents a bias term and $\mathrm{f}$ is the concatenated original fingerprint. We use the reduced dimensional fingerprint ${\mathrm{f}}^{\prime }$ as the molecular fingerprint feature.

Finally, the molecular substructure representation module outputs the molecular subgraph features and the molecular fingerprint features for subsequent feature fusion.

#### Multi-source information fusion and predictive module

##### Attention interaction between molecular graph-level representation and subgraph features

To interrogate the connection between molecular subgraph features and graph-level representations, we developed a cross-scale attention interactive mechanism that combines subgraph feature representations with graph-level feature representations, and then we obtain more effective subgraph feature representations. Specifically, we employ a graph attention network (GAT) [41] to compute weights between molecular subgraph features and their corresponding molecular graph-level representations through a self-attention mechanism. This enables the fusion of local topological information from the molecular graph with global topological information, resulting in more accurate molecular subgraph features.

Given a graph-level representation m of a molecule, the k^th^ subgraph feature corresponding to m is denoted as ${\mathrm{s}}_{\mathrm{k}}$. We firstly compute the association vector between m and ${\mathrm{s}}_{\mathrm{k}}$, denoted as ${\mathrm{e}}_{\mathrm{k}}$ (Eq. 10).


(10) \begin{equation*} {\mathrm{e}}_{\mathrm{k}}=\mathrm{LeakyReLU}\left({\mathrm{a}}^{\top}\left[{\mathrm{W}}_{pro}{\mathrm{s}}_{\mathrm{k}}\Big\Vert{\mathrm{W}}_{pro}\mathrm{m}\right]\right) \end{equation*}

Here, ${\mathrm{W}}_{pro}$ represents the linear projection matrix, a is the attention vector, and ‖ denotes vector concatenation, LeakyReLU denotes the Leaky Rectified Linear Unit activation function. Secondly, we perform softmax [42] normalization on all attention vectors corresponding to m (Eq. 11)


(11) \begin{equation*} {\mathrm{a}}_{\mathrm{k}}=\frac{\exp \left({\mathrm{e}}_{\mathrm{k}}\right)}{\sum_{\mathrm{u}\in \mathrm{S}}\kern0.20em \exp \left({\mathrm{e}}_{\mathrm{u}}\right)} \end{equation*}

where S represents the set of subgraphs corresponding to m.

Finally, we weight the subgraph features using attention weights to have the updated representation of the molecular subgraph features (Eq. 12)


(12) \begin{equation*} {\mathrm{s}}_{\mathrm{m}}=\mathrm{ReLU}\left(\sum_{\mathrm{u}\in \mathrm{S}}\kern0.1em {\mathrm{a}}_{\mathrm{k}}{\mathrm{W}}_k{\mathrm{s}}_{\mathrm{k}}\right) \end{equation*}

Here, ${\mathrm{W}}_{\mathrm{k}}$ is a learnable matrix.

##### Multi-source feature fusion

To obtain the final molecular representation for prediction, we fused molecular graph-level representations, molecular subgraph features, and molecular fingerprint features together. Specifically, we concatenated these three types of information and used an MLP (multilayer perceptron [43, 44]) to transform them into the final molecular representation P (Eq. 13).


(13) \begin{equation*} \mathrm{P}=\mathrm{mlp}\left(\mathrm{m}\right\Vert\ {\mathrm{s}}_{\mathrm{m}}\mid \left|{\mathrm{f}}^{\prime}\right) \end{equation*}

Here, m represents the molecular graph representation, ${\mathrm{s}}_{\mathrm{m}}$ represents the molecular subgraph feature, and ${\mathrm{f}}^{\prime }$is the molecular fingerprint feature.

##### Comparison and analysis of prediction

Firstly, we performed predictions on the eight commonly used molecular property predictive datasets mentioned in *Dataset* Section. Secondly, we employed receiver operating characteristic–area under the curve (ROC-AUC) [45] and root mean square error (RMSE) [46] to evaluate classification and regression tasks [47–51], respectively. Lastly, we selected eleven baseline models (Supplementary Table S3) for performance comparison and analysis.

#### Web server development

For the backend, we employ Python and Flask [52] as the development frameworks. Combined with scientific computing and data analysis libraries such as Pandas [53] and NumPy [54], we perform data cleaning, statistical analysis, and feature extraction on molecular related datasets. On the front end, we build up page structures and interactive logic using HyperText Markup Language (HTML), Cascading Style Sheets (CSS), and native JavaScript. Additionally, we employ ECharts [55] to make charts like molecular feature distributions, thereby intuitively presenting datasets and visualizing model prediction results.

## Results

To answer each scientific question, we not only conducted experimental and comparative analyses for the overall predictive performance of the MSF-HierGNN model, but also developed an ablation study and the visualization methods to demonstrate the effectiveness for each module.

### Predictive performance and comparative analysis of MSF-HierGNN

**Figure 3:**
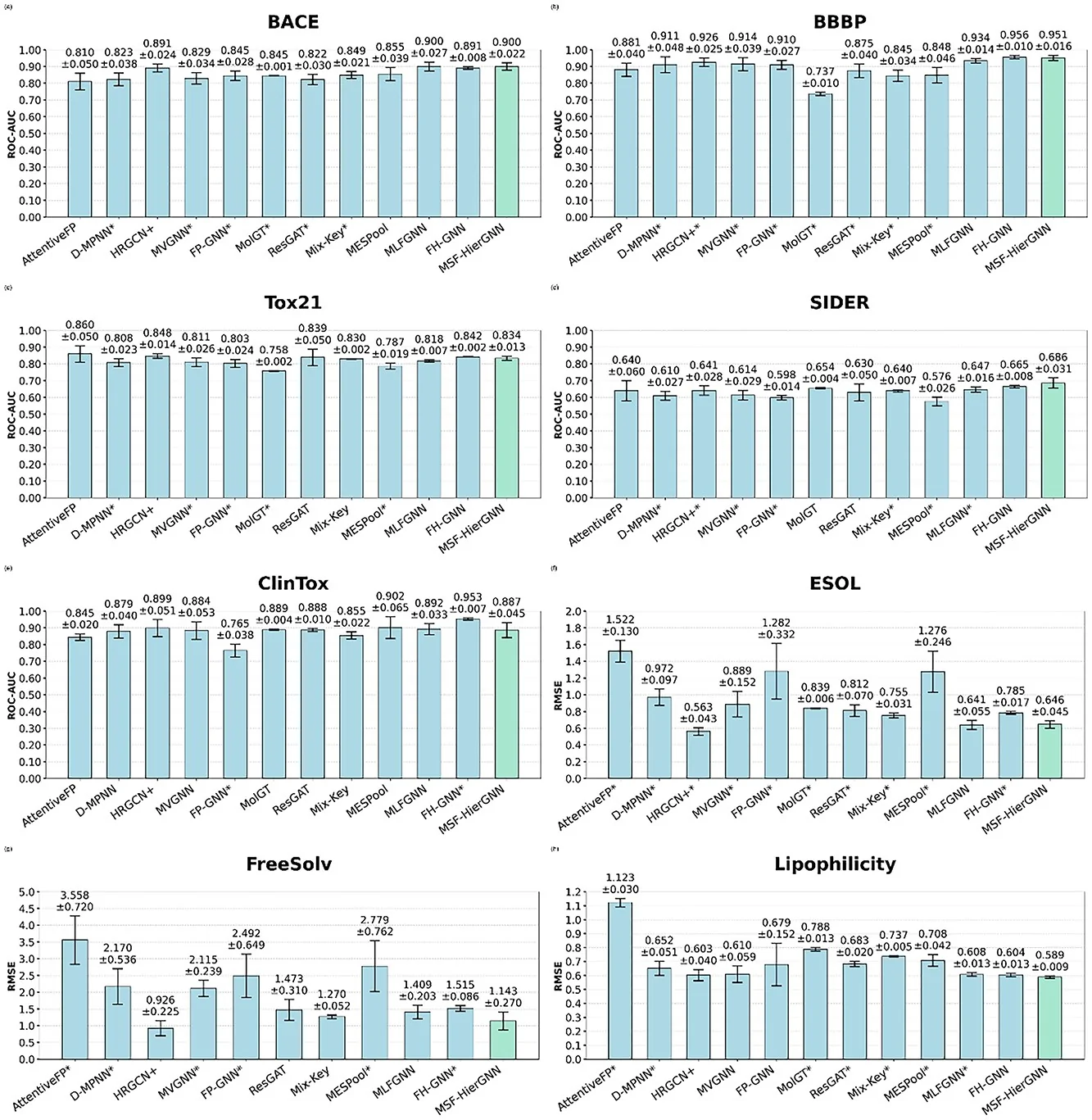
Predictive performance of different models on 8 datasets. The * symbol indicates that the results are statistically significantly different (*P*-values ≤ .05) from our MSF-HierGNN model calculated by T-test.

To address our first scientific question, we designed a comparative experiment to validate if our MSF-HierGNN model can achieve better predictive performance than 11 baseline models (Supplementary Table S3), after it obtains a more comprehensive molecular graph-level representation. The results are shown in Fig. 3.

Fig. 3 demonstrate that the predictive accuracy of MSF-HierGNN is statistically significantly better than the most of the eleven baseline models on four datasets: BACE, BBBP, SIDER, and Tox21 on the five classification task datasets. For example, on the BBBP dataset, MSF-HierGNN is statistically significantly better than eight baseline models such as D-MPNN and HRGCN+, and statistical nonsignificant for the remaining two models. On the ClinTox dataset, MSF-HierGNN only statistically significantly outperformed the FP-GNN model, and showing statistically nonsignificant compared to other nine baseline models.

MSF-HierGNN statistically significantly better than seven out of eleven baseline models on three regression task datasets, and showing statistically nonsignificant compared to other models on both the Lipophilicity and FreeSolv datasets. On the ESOL dataset, the performance of MSF-HierGNN is statistically significantly better than eight out of eleven baseline models, but it is statistically significantly less than HRGCN+ model.

In summary, the performance and comparative analysis demonstrates that MSF-HierGNN can increase predictive accuracy by providing comprehensive extraction of molecular substructures.

### Ablation study

To answer our second scientific question, we design the ablation study by removing or replacing a specific module within the model. And then, we compare it with the complete model to validate the effectiveness of different modules of the complete model.

Here, we removed the Structure-Aware Molecular Substructure Representation Module (*Molecular Substructure Representation Module* Section) and the Structure-Aware Hierarchical Pooling Module (*Structure Aware Hierarchical Pooling Module for Molecular Graphs* Section) from MSF-HierGNN to observe the impact of these two modules on the final prediction.

**Figure 4:**
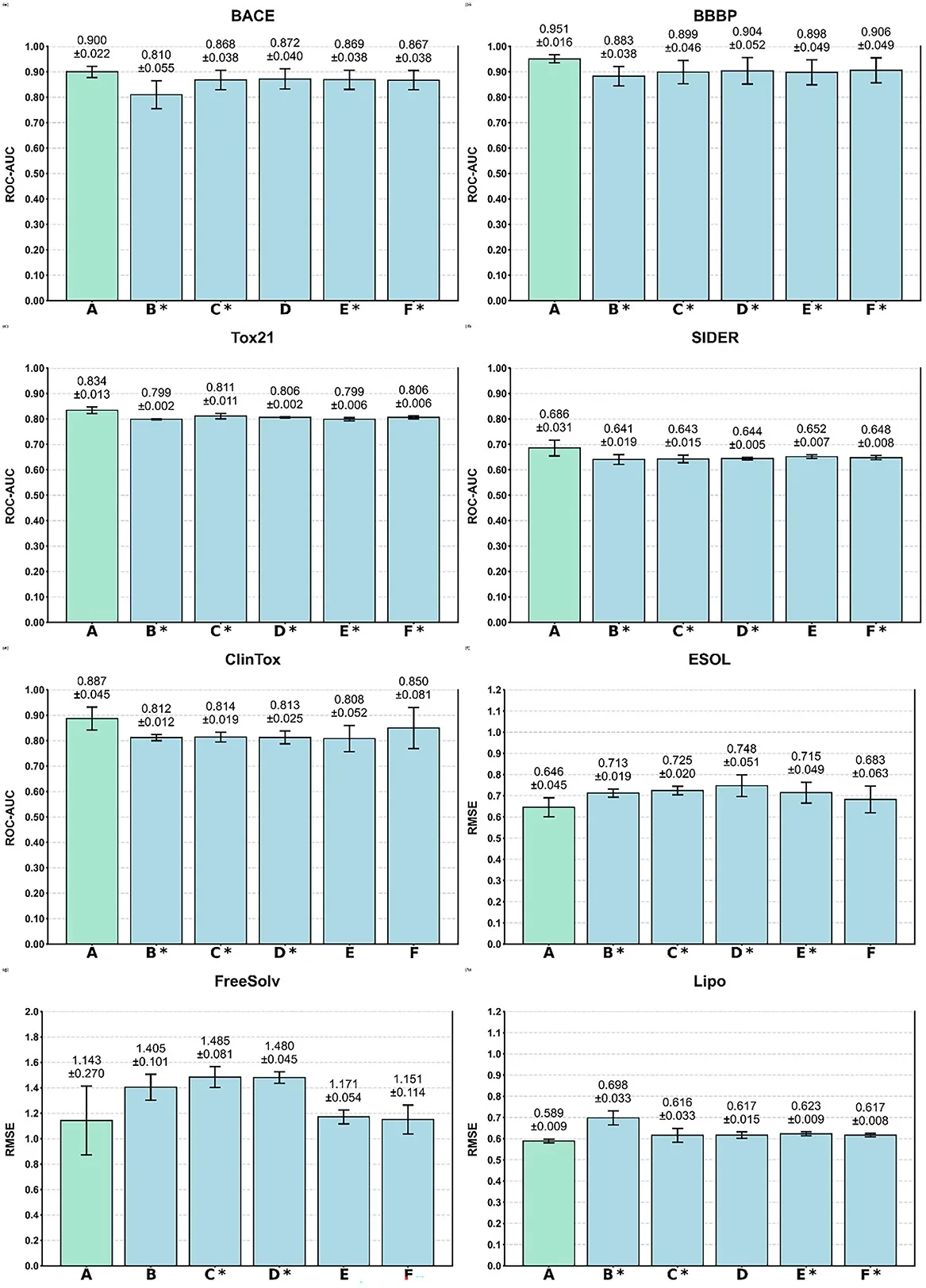
Ablation study results. Here, (a–f) on the horizontal axis represent (a) Full MSF-HierGNN model, (b) Missing the structure-aware molecular substructure representation module, (c) Missing the structure-aware hierarchical pooling module, (d) Replace the message passing operator with GCN, (e) Replace the message passing operator with DMPNN and (f) Replace the message passing operator with GIN, respectively. The * symbol indicates that the results are statistically significantly different (*P*-values ≤ .05) from the full MSF-HierGNN model calculated by T-test.

Additionally, we replaced the message passing operators in *Multi-source Information Fusion and Predictive Module* Section with GCN [19], DMPNN [20], and GINE [56]. The results of the ablation study are shown by Fig. 4.

As shown in Fig. 4, when the Structure-Aware Molecular Substructure Representation Module is missing, the ROC-AUC values across five classification task datasets are statistically significantly less than those of the complete model. On two of the three regression task datasets, the RMSE is statistically significantly greater than that of the complete model, but the FreSolv dataset shows statistically non-significance.

After missing the Structure-Aware Hierarchical Pooling Module (model using the global graph pool), the ROC-AUC on five classification task datasets is statistically significantly less than that of the complete model, which employs the Hierarchical Graph Pooling Module with a maximum reduction from 0.887 to 0.814 (Fig. 4c, on the tox21 dataset). Conversely, the RMSE values across three regression task datasets are statistically significantly greater than those of the complete model (Fig. 4f–h) with the maximum increasing from 1.143 to 1.485 (Fig. 4g, on the freesolv dataset).

And no matter whether we replace the message passing operator in the Multi-source Information Fusion and Predictive Module with GCN, DMPNN, or GINE, the ROC-AUC is statistically significantly less than that of the complete model on the five classification task datasets. On the three regression task datasets, except for a few cases, such as when the operator is replaced with GIN on the FreeSolv dataset, the results were not statistically significant. In other cases, the RMSE is statistically significantly greater than that of the complete model.

The results of the ablation study above demonstrate that removing any of these modules from the model will significantly impact its predictive accuracy. This also turns out the effectiveness of our innovation in Structure-Aware Molecular Substructure Representation Module, Structure-Aware Hierarchical Pooling Module, and the message passing.

### Visualization and comparison analysis of atomic feature correlations

**Figure 5:**
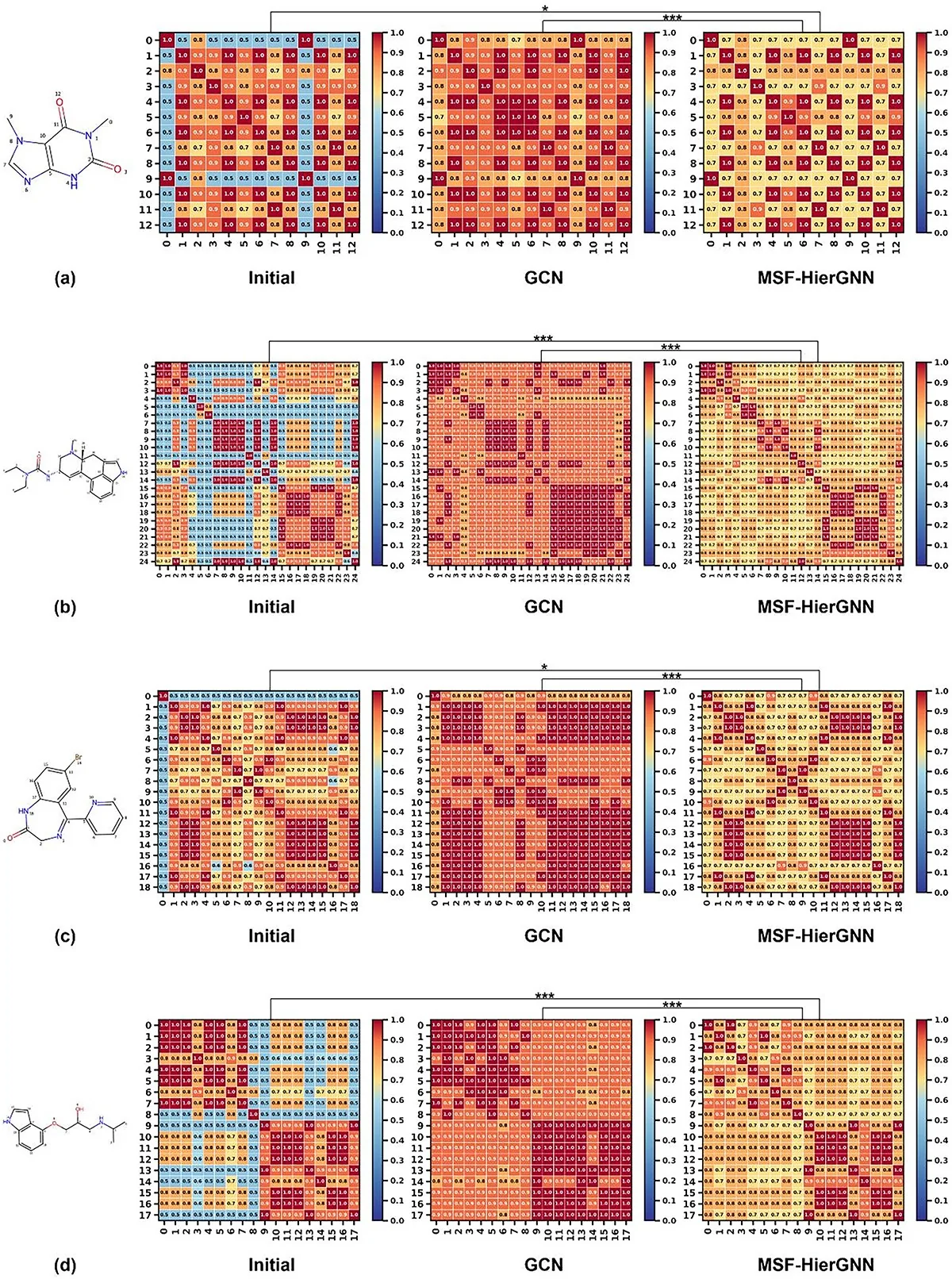
Visual comparison of atomic feature correlations in molecules from classification task data. Subfigures (a), (b), (c), and (d) represent four randomly selected molecules from the classification task dataset. Each subfigure displays, from left to right: The molecular chemical structure; the initial state; the atomic cosine similarity heatmap after classical GCN message passing; and the atomic cosine similarity heatmap after our MSF-HierGNN message passing. ‘*’ indicates statistically significant differences calculated by T-test. ‘*’, ‘**’, and ‘***’ denote *P*-values ≤ .05, *P*-values ≤ .01, and *P*-values ≤ .001, respectively.

**Figure 6:**
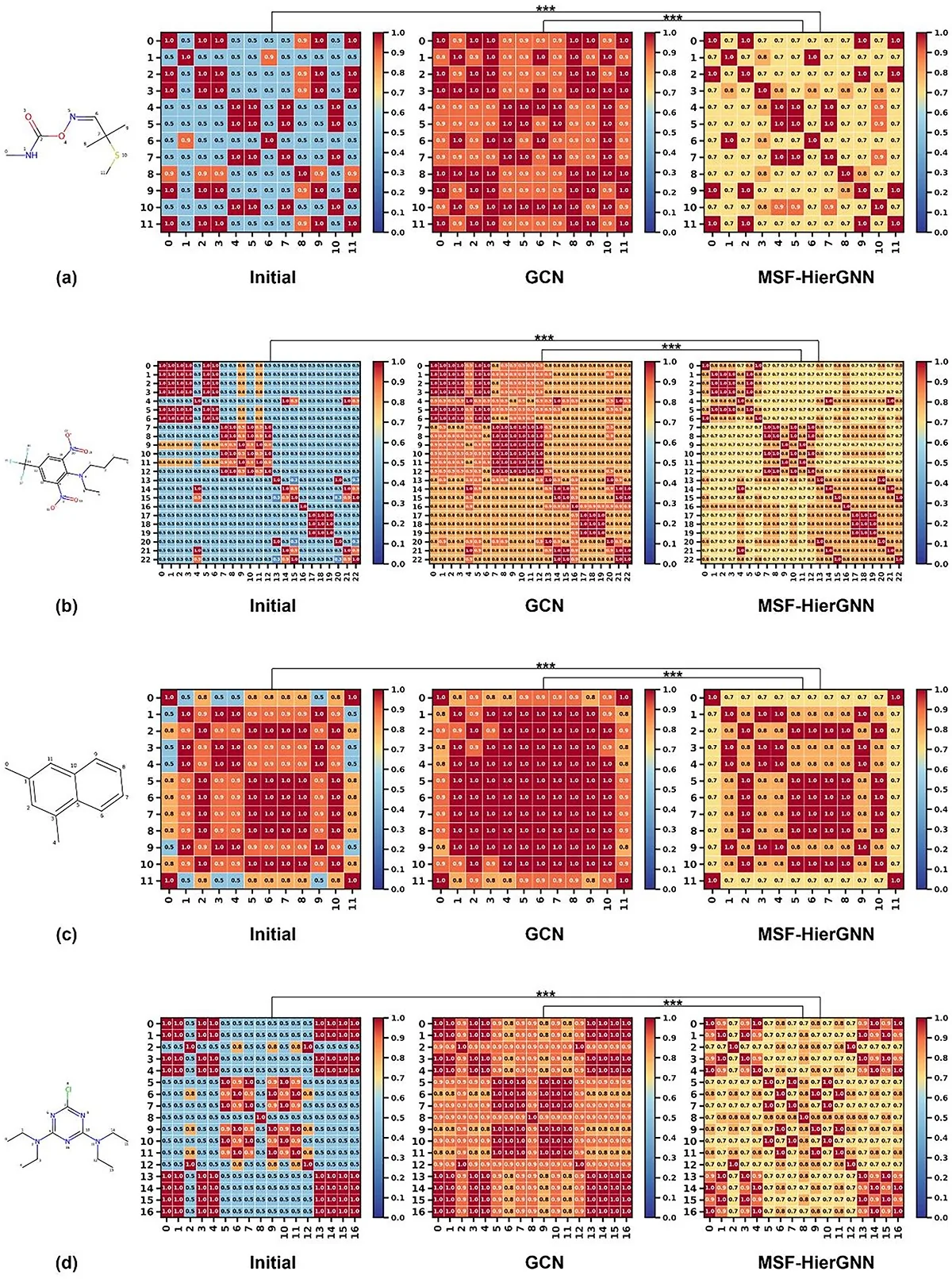
Visual comparison of atomic feature correlations in molecules from regression task data.

For the third scientific question of this study, we performed a comparison analysis of correlations among atomic features under different states by heatmaps to demonstrate the effectiveness of the modified MSF-HierGNN message passing. Since cosine similarity can reflect the degree of similarity among atomic features, we randomly selected four molecules from five classification task datasets and three regression task datasets respectively at first. Then, we consecutively computed the cosine similarity between atomic features for these molecules in their initial state process, after classical GCN message passing process, and after MSF-HierGNN message passing process. Finally, we performed a heatmap visualization comparison of cosine similarity under different conditions and conducted statistical tests to analyze the differences. The results are shown in Figs. 5 and 6.

First of all, Figs 5 and 6 demonstrate that the atomic cosine similarity after MSF-HierGNN message passing process is statistically significant different from the atomic cosine similarity from initial state process and after GCN message passing process. And this pattern holds true for both the molecules in classification task and regression task data.

Secondly, since the cosine similarity in the initial state process has a similar color with after GCN message passing process across heatmaps, it indicates that GCN does not consider the differential importance of atomic chemical bonds in the message passing process, which cannot reflect the correlations among atomic features.

And the heatmaps employing MSF-HierGNN message passing obviously highlight the differences of atomic chemical bonds. For example, Fig. 5d shows since the atoms with index 0, 1, and 2 are all carbon atoms, the cosine similarity among them is initially 1. However, after MSF-HierGNN message passing, because atom 1 is connected to three atoms whereas atoms 0 and 2 are only connected to atom 1, the cosine similarity between atom 0 and atom 2 is still 1, but the cosine similarity between atoms 0 & 2 and atom 1 is reduced to 0.8, which correctly reflects the atomic features of local structural patterns.

In summary, the aforementioned results demonstrate that the improved message passing mechanism of the MSF-HierGNN model can capture the correlations among atomic features.

### MSF-HierGNN web server platform

**Figure 7:**
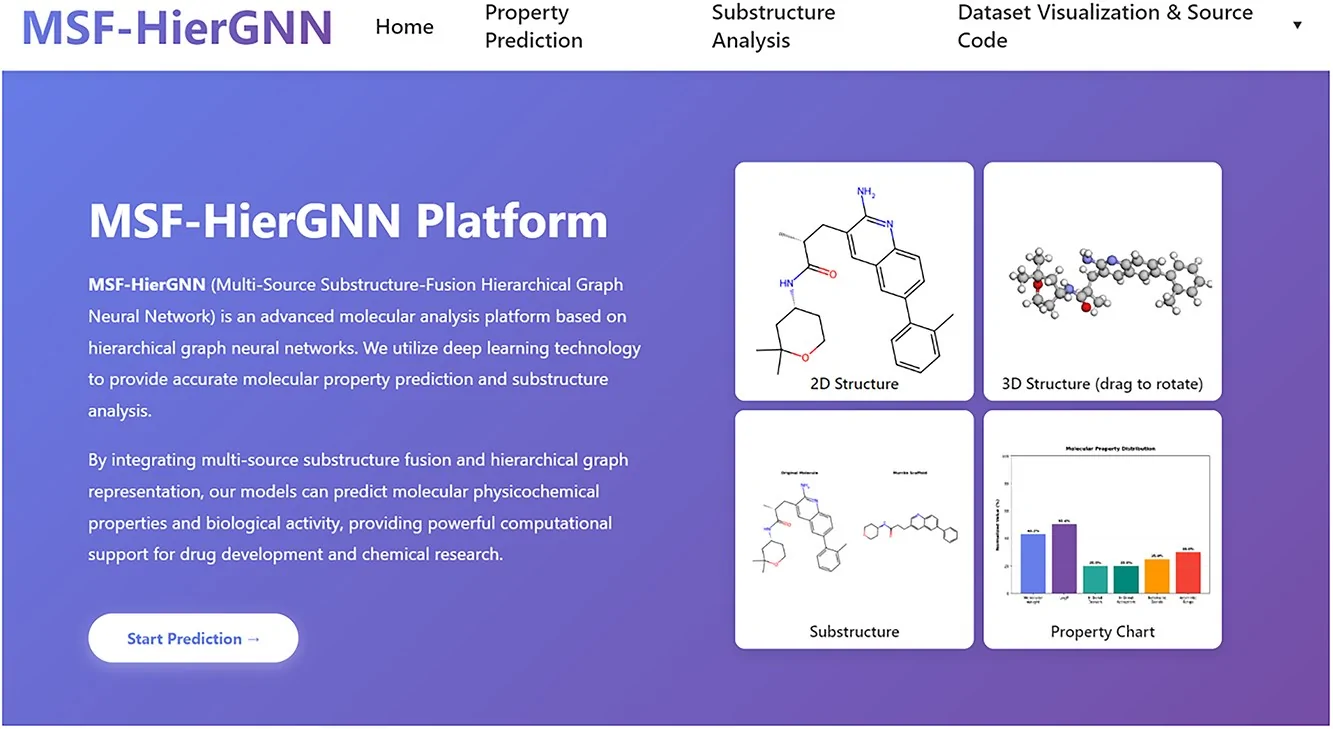
The homepage of MSF-HierGNN online platform.

The homepage (www.combio-lezhang.online/MSF-HierGNN/) of MSF-HierGNN online platform is shown in Fig. 7 and Supplementary Fig. S3 with three key modules.

First is Property Prediction Module (Supplementary Fig. S4). After the user inputs a SMILES string or selects a molecule from the dropdown list, the platform predicts its 2D/3D molecular structure, property distributions, and property prediction results.

Second is Substructure Analysis Module (Supplementary Fig. S5). Users can employ this module to obtain visualized results for molecular substructure information, including BRICS fragments, Murcko skeletons, and five types of molecular fingerprints (*Molecular Substructure Representation Module* Section).

Third is Dataset Visualization Module (Supplementary Fig. S6). This module not only enables users to query feature distribution charts and data quality reports for eight benchmark datasets (*Dataset* Section, Table S1), but also offers code download functionality for the MSF-HierGN model.

To further evaluate the practical performance of the web server, we tested its response speed, stability, and concurrent access capability (Supplementary Table S4). The results show that the average response time of the system is 23–28 ms under low concurrent load, and the maximum response time is ~1.9 s under concurrent and multi request load, indicating that the system function is stable.

## Discussion and conclusion

To address the limitations of existing graph neural networks to predict drug molecular properties, including incomplete representation of molecular hierarchical structures, insufficient exploration of local topological features, and under-extraction of correlations among atomic features, this study proposes a graph neural network that combines hierarchical pooling with localized substructure modeling named MSF-HierGNN (Multi-Source Substructure-Fusion Hierarchical Graph Neural Network).

In terms of the overall predictive performance of the model, the classification criteria of the ACE, BBBP, and SIDER datasets are closely associated with specific molecular substructures or features [57–59], and MSF-HierGNN achieved the statistically significantly best optimal predictive effect across all three datasets (Fig. 3a–e) for classification tasks. Thus, it demonstrates that MSF-HierGNN can increase predictive accuracy by providing comprehensive extraction of molecular substructures.

On the other two classification task datasets, Tox21 and ClinTox, MSF-HierGNN achieved superior predictive performance but did not reach the highest accuracy (the highest being Attentive FP at 0.860 ± 0.050 and MESPool at 0.902 ± 0.065). Similar to MSF-HierGNN, FP-GNN, and MLFGNN employ molecular fingerprinting to augment molecular substructures. And those two methods demonstrated unfavorable predictive accuracy on datasets Tox21 and ClinTox. This may be caused by the heavy data imbalance in the Tox21 [60] and the fact that the classification criteria for the ClinTox are not entirely dependent on chemical structure, introducing certain biases [61].

For regression tasks (Fig. 3f–h), we observed that MSF-HierGNN achieved the statistically significantly best predictive accuracy across all three regression datasets, though it did not receive the best performance on ESOL and FreeSolv. We consider the reason as: molecular properties such as solubility, solvation free energy, and water partition coefficient are often determined by the overall physicochemical properties of the molecule and its interactions with the solvent, rather than being directly associated with specific substructures [4, 7, 62].

Overall, Fig. 3 demonstrate that MSF-HierGNN can not only effectively extract molecular substructure features, but also more accurately predict molecular properties to guide the design of drug molecules targeting specific proteins.

In terms of ablation study, Fig. 4 indicates that after removing the Molecular Substructure Representation Module, the performance of the model statistically significantly declines across all five classification task datasets. This not only validates the effectiveness of the Molecular Substructure Representation Module, but also further indicates that molecular classification is closely associated with specific substructures or features.

Secondly, removing the Structure-Aware Hierarchical Pooling Module from the molecular graph structure-aware layer leads to statistically significant performance declines across all eight datasets. This demonstrates that the hierarchical molecular graph representation we proposed can extract more enriched molecular features, thereby increasing the accuracy of molecular property predictions.

Additionally, after replacing the message passing which carries chemical bond information with classical GCN, DMPNN, and GIN, the model performance exhibited a statistically significant decline, but it was less pronounced than when the first two modules were removed. This may be the molecular information extracted by the Molecular Substructure Representation Module and the Structure-Aware Hierarchical Pooling Module already encompassed a certain degree of chemical bond information [20].

Finally, Fig. 4g shows that the model's performance on the FreeSolv dataset does not statistically significantly decline enough after module removing or replacement (only 2 out of 5 studies show statistically significant differences). This may be attributed to FreeSolv having the smallest sample size among the eight datasets (only 642 molecules, shown in Table S1).

In terms of visualization comparisons of atomic feature correlations, Figs 5 and 6show that the classical GCN tends to homogenize neighborhood atomic information during message passing, which makes the cosine similarity heatmaps appear overly smoothed and lack local recognition [63]. Unlike that, the cosine similarity heatmaps after message passing of MSF-HierGNN show more distinct contrasts, presenting statistically significant differences among atoms with different topological structures. Since chemical bonds typically possess semantically rich features [64], explicitly incorporating this edge information into the message passing process enables the model to better represent important local structural patterns. These local molecular structural patterns not only further increase the accuracy of property predictions but also help us to prevent drug design failures caused by conformational mismatches or off-target effects at a later stage.

Although MSF-HierGNN achieved performance improvements in molecular property predictive tasks, there are several problems that need to be optimized. For example, the model still has limitations in extracting three-dimensional features of molecules and molecular videos [65, 66], and fast, user-friendly predictive online services have yet to be established. Therefore, we firstly plan to further increase the accuracy of molecular property predictions by using pretraining large models [67, 68] to capture atoms-level interactions in three-dimensional space. Secondly, to fully leverage the vast amounts of unlabeled data in drug molecule databases [69, 70], we should speed up model training for downstream tasks by employing pre-training algorithms [71], to make the model better adapt to the features of different molecular datasets. Finally, we need develop a high-performance computing [47, 72] based visual online application to foster a faster and more user-friendly web-server platform [73] for drug molecule property prediction.

**Key Points**

- By progressively filtering important atoms layer by layer in the molecular graph, MSF-HierGNN can extract more comprehensive hierarchical information from the graph, thereby generating more accurate graph-level representation.
- MSF-HierGNN introduces five molecular fingerprints to capture chemically meaningful substructures, thereby offering a more comprehensive representation for molecular substructure features.
- MSF-HierGNN performs weighted aggregation of these features to increase its ability to distinguish the importance of different chemical bonds and the predictive accuracy.
- This study develops an interactive, user-friendly web server application for visualizing and analyzing molecular property predictions based on MSF-HierGNN algorithm.

## Supplementary Material

Supplementary_Information06142026_bbag491

## Data Availability

The source code of the MSF-HierGNN algorithm, can be found in https://github.com/flymetothestar11/MSF-HierGNN-Source-Code.See  Supplementary Information for more details of methods and results.
