# Supplementary material for: MSF-HierGNN: a multi-source substructure-fusion hierarchical GNN method and web server to predict molecular property for drug design
Source: Brief Bioinform. 2026 Sep 10;27(5):bbag491. doi: 10.1093/bib/bbag491 (PMC13561308; doi:10.1093/bib/bbag491)
Supplement: Supplementary file 1 [file supplementary_information06142026_bbag491.docx]

**SUPPLEMENTARY INFORMATION**

**Supplementary Tables**

**Table S1**. Summary of eight benchmark datasets

**Table S2.** Atom features and Bond features

**Table S3.** Baseline Models Summary Table

**Table S4.** Website testing Summary Table

**Supplementary Figures**

**Figure S1.** The process of Structure Aware Hierarchical Pooling Module for Molecular Graphs

**Figure S2.** The process of Molecular Substructure Representation Module

**Figure S3.** The homepage of MSF-HierGN online platform

**Figure S4.** The Property Prediction page of MSF-HierGN online platform

**Figure S5.** The Substructure Analysis Model page of MSF-HierGN online platform

**Figure S6.** The Dataset Visualization page of MSF-HierGN online platform

**Supplementary Tables**

**Table S1.** Summary of eight benchmark datasets

| **Dataset** | **Task type** | **#Tasks** | **#Molecules** | **Avg nodes/molecule** | **Split strategy** | **Metric** | **Description** | **Label definition** |
| --- | --- | --- | --- | --- | --- | --- | --- | --- |
| BACE | Classification | 1 | 1513 | 34 | Scaffold split | ROC-AUC | **Binary classification dataset for inhibitory activity against human beta-secretase 1 (BACE-1).** | Whether a compound is an active BACE-1 inhibitor. |
| BBBP | Classification | 1 | 2039 | 24 | Scaffold split | ROC-AUC | **Binary classification dataset related to blood-brain barrier penetration.** | Whether a compound can penetrate the blood-brain barrier. |
| Tox21 | Classification | 12 | 7831 | 19 | Random split | ROC-AUC | **Toxicity dataset from the Tox21 program.** | Includes 12 toxicity tasks; for each task, the label indicates whether the assay outcome is positive. |
| SIDER | Classification | 27 | 1427 | 34 | Random split | ROC-AUC | **Dataset of marketed drugs and their adverse drug reactions.** | Multi-label adverse reaction indicators (present = 1, absent = 0). |
| ClinTox | Classification | 2 | 1478 | 26 | Random split | ROC-AUC | **Binary classification dataset comparing FDA-approved drugs with drugs that failed clinical trials due to toxicity.** | Two labels: (1) whether the drug failed clinical trials due to toxicity; (2) whether the compound is FDA-approved. |
| ESOL | Regression | 1 | 1128 | 13 | Random split | RMSE | **Regression dataset of small-molecule aqueous solubility.** | Experimentally measured solubility value. |
| FreeSolv | Regression | 1 | 642 | 9 | Random split | RMSE | **Dataset of hydration free energies of small molecules in water.** | Experimentally measured hydration free energy. |
| Lipophilicity | Regression | 1 | 4200 | 27 | Random split | RMSE | **Regression dataset related to the octanol/water partition coefficient.** | Experimentally measured partition coefficient (lipophilicity). |

**Table S2.** Atom features and Bond features

| **Feature** | **#Categories** | **Description** |
| --- | --- | --- |
| Atom type | 60 | Atomic number (element type). |
| Degree | 7 | Number of directly connected neighbors. |
| Hybridization | 6 | Hybridization type of the atom. |
| Total H count | 6 | Total number of hydrogens attached to the atom. |
| Chirality | 3 | Stereochemistry category of the atom. |
| Formal charge | 1 | Formal charge value. |
| Radical electrons | 1 | Number of radical electrons. |
| Aromaticity | 1 | Whether the atom is aromatic (yes = 1, no = 0). |
| H-bond donor | 1 | Whether the atom is a hydrogen-bond donor (yes = 1, no = 0). |
| H-bond acceptor | 1 | Whether the atom is a hydrogen-bond acceptor (yes = 1, no = 0). |
| Acidic | 1 | Whether the atom belongs to an acidic group (yes = 1, no = 0). |
| Basic | 1 | Whether the atom belongs to a basic group (yes = 1, no = 0). |
| Halogen | 1 | Whether the atom is a halogen (yes = 1, no = 0). |
| Murcko scaffold | 1 | Whether the atom belongs to the Murcko scaffold (yes = 1, no = 0). |
| Bond type | 4 | Chemical bond type. |
| Stereochemistry type | 4 | Bond stereochemistry type. |
| Conjugated | 1 | Whether the bond is conjugated (yes = 1, no = 0). |
| In ring | 1 | Whether the bond is in a ring (yes = 1, no = 0). |
| Interatomic distance | 1 | Distance between the two atoms. |

**Table S3.** Baseline Models Summary Table

| **No.** | **Name** | **Year** | **Journal** | **Description** |
| --- | --- | --- | --- | --- |
| 1 | AttentiveFP [1] | 2019 | Journal of medicinal chemistry | combines graph convolution layers with an attention mechanism for molecular property prediction. |
| 2 | D-MPNN [2] | 2019 | Journal of chemical information and modeling | uses directed-bond-associated messages rather than atom-associated messages to avoid unnecessary loops in message passing. |
| 3 | HRGCN+ [3] | 2021 | Briefings in bioinformatics | takes both molecular descriptors and molecular graphs as inputs to better capture hierarchical structural features and improve prediction performance. |
| 4 | MVGNN [4] | 2022 | Bioinformatics | introduces a cross-dependent message passing scheme to enhance model expressiveness. |
| 5 | FP-GNN [5] | 2022 | Briefings in bioinformatics | jointly learns information from molecular graphs and fingerprints for molecular property prediction. |
| 6 | MolGT [6] | 2025 | Information Fusion | proposes a multimodal pretraining framework for molecular representations that integrates graph structure, SMILES sequences, and domain knowledge with a unified graph-transformer architecture. |
| 7 | ResGAT [7] | 2024 | Memetic Computing | introduces a residual graph attention network that combines multi-head attention with residual connections to strengthen information propagation in molecular graphs. |
| 8 | Mix-Key [8] | 2024 | Briefings in bioinformatics | uses data augmentation to generate more informative molecular graphs, improving molecular representations. |
| 9 | MESPool [9] | 2024 | Briefings in Bioinformatics | proposes a bond-contraction-based molecular graph pooling method that helps retain key chemical structures. |
| 10 | MLFGNN [10] | 2025 | Journal of Chemical Information and Modeling | fuses molecular graph information with molecular fingerprint information to capture both global and local molecular features. |
| 11 | FH-GNN[11] | 2025 | Journal of Pharmaceutical Analysis | constructs hierarchical molecular graphs by integrating atom-level, motif-level, and graph-level information, and combines them with molecular fingerprints using an adaptive attention mechanism for molecular property prediction. |

**Table S4.** Website testing Summary Table

| **test** | | | **concurrency** | **requests** | | **success_percent** | | **requests_per_second** | | **mean_ms** | | | **p95_ms** | **p99_ms** | | **max_ms** | | **failed** | |  |
| --- | --- | --- | --- | --- | --- | --- | --- | --- | --- | --- | --- | --- | --- | --- | --- | --- | --- | --- | --- | --- |
|  | response_speed | 1 | | | 30 | | 100.0 | | 43.86 | | 22.69 | 34.73 | | | 42.11 | | 43.95 | | 0 | |
|  | concurrent_access_1 | 1 | | | 20 | | 100.0 | | 44.18 | | 22.56 | 38.63 | | | 41.58 | | 42.32 | | 0 | |
|  | concurrent_access_5 | 5 | | | 20 | | 100.0 | | 197.45 | | 23.77 | 26.20 | | | 27.52 | | 27.85 | | 0 | |
|  | concurrent_access_10 | 10 | | | 20 | | 100.0 | | 313.15 | | 27.71 | 32.02 | | | 32.21 | | 32.25 | | 0 | |
|  | concurrent_access_50 | 50 | | | 200 | | 100.0 | | 173.5 | | 149.3 | 413.9 | | | 1038.57 | | 1059.16 | | 0 | |
|  | concurrent_access_100 | 100 | | | 200 | | 100.0 | | 99.6 | | 351.2 | 1036.37 | | | 1315.43 | | 1896.51 | | 0 | |
|  | stability | 3 | | | 100 | | 100.0 | | 38.36 | | 77.26 | 100.48 | | | 127.95 | | 128.12 | | 0 | |
|  | stability | 100 | | | 100 | | 100.s0 | | 359.36 | | 193.3 | 231.23 | | | 234.43 | | 235.27 | | 0 | |

**Supplementary Figures**

**Figure S1.** The process of Structure Aware Hierarchical Pooling Module for Molecular Graphs.

**Figure S2.** The process of Molecular Substructure Representation Module

**Figure S3.** The homepage of MSF-HierGN online platform. The Home page displays example visualizations (2D/3D molecular structures, substructure analysis, property charts) and provides navigation buttons to access these three functional modules.

**Figure S4.** The Property Prediction page of MSF-HierGN online platform. After the user inputs a SMILES string or selects a molecule from the dropdown list, the platform predicts its 2D/3D molecular structure, property distributions, and property prediction results.

**Figure S5.** The Substructure Analysis Model page of MSF-HierGN online platform. Users can employ this module to obtain visualized results for molecular substructure information, including BRICS fragments, Murcko skeletons, and five types of molecular fingerprints.

**Figure S6.** The Dataset Visualization page of MSF-HierGN online platform. This page not only enables users to query feature distribution charts and data quality reports for eight benchmark datasets, but also provides code download functionality for the MSF-HierGN model.

**References**

[1] Z. Xiong, D. Wang, X. Liu, et al., Pushing the boundaries of molecular representation for drug discovery with the graph attention mechanism, Journal of Medicinal Chemistry 63(16) (2019) 8749-8760.

[2] K. Yang, K. Swanson, W. Jin, et al., Analyzing learned molecular representations for property prediction, Journal of Chemical Information and Modeling 59(8) (2019) 3370-3388.

[3] Z. Wu, D. Jiang, C.-Y. Hsieh, G. Chen, B. Liao, D. Cao, T.J.B.i.b. Hou, Hyperbolic relational graph convolution networks plus: a simple but highly efficient QSAR-modeling method, 22(5) (2021) bbab112.

[4] H. Ma, Y. Bian, Y. Rong, W. Huang, T. Xu, W. Xie, G. Ye, J.J.B. Huang, Cross-dependent graph neural networks for molecular property prediction, 38(7) (2022) 2003-2009.

[5] H. Cai, H. Zhang, D. Zhao, et al., FP-GNN: a versatile deep learning architecture for enhanced molecular property prediction, Briefings in Bioinformatics 23(6) (2022) bbac408.

[6] R. Chen, C. Li, L. Wang, M. Liu, S. Chen, J. Yang, X.J.I.F. Zeng, Pretraining graph transformer for molecular representation with fusion of multimodal information, 115 (2025) 102784.

[7] T.-H. Nguyen-Vo, T.T. Do, B.P.J.M.C. Nguyen, ResGAT: Residual Graph Attention Networks for molecular property prediction, 16(3) (2024) 491-503.

[8] T. Jiang, Z. Wang, W. Yu, J. Wang, S. Yu, X. Bao, B. Wei, Q.J.B.i.B. Xuan, Mix-Key: graph mixup with key structures for molecular property prediction, 25(3) (2024).

[9] F. Xu, Z. Yang, L. Wang, D. Meng, J.J.B.i.B. Long, MESPool: Molecular Edge Shrinkage Pooling for hierarchical molecular representation learning and property prediction, 25(1) (2023).

[10] X. Liu, C. Fan, Y. Liu, H.-b.J.J.o.C.I. Li, Modeling, Multilevel Fusion Graph Neural Network for Molecule Property Prediction, (2025).

[11] Liu, S., et al. Fingerprint-enhanced hierarchical molecular graph neural networks for property prediction. Journal of Pharmaceutical Analysis 2025;15(6):101242.
